# Poisoning by Green Paper-Hangings

**Published:** 1874-03

**Authors:** 


The Rev. J. M. Drake and wife, of Lima, Ohio, have just died from poisoning. They were preparing to paper a room in their house, and in tearing down the old paper, which was of a deep green color, dust was created, which was inhaled by them, and they were poisoned by it. They died within a short time of each other, and were buried on the same day.

